# Investigation of a ^47^Sc-radiolabelled PDGFRβ-targeted affibody in SPECT imaging and radiotherapy for pancreatic cancer

**DOI:** 10.1186/s12885-025-15506-w

**Published:** 2026-01-05

**Authors:** Ruomeng Liu, Dongping Su, Yuhao Liao, Zhao Li, Bo Li, Yunming Chen, Qi Cao, Jinsong Zhang, Huawei Cai

**Affiliations:** 1 Department of Nuclear Medicine & Laboratory of Clinical Nuclear Medicine, West China Hospital, Sichuan University, Chengdu, 610041 China; 2 The First Sub-institute of Nuclear Power Institute of China, Chengdu, 610005 China; 3 National Engineering Research Center of Isotopes and Medicine, Chengdu, 610213 China; 4 Sichuan Provincial Engineering Research Center of Radiopharmaceutical Clinical Translation, Sichuan University, Chengdu, Sichuan 610041 China

**Keywords:** Pancreatic cancer, Platelet derived growth factor receptor beta, Affibody, ^47^Sc, SPECT/CT, Radionuclide therapy

## Abstract

Pancreatic cancer is a malignant solid tumour that contains a large number of cancer-associated fibroblasts (CAFs). Therefore, evaluating tumour disease progression and planning radionuclide therapy using molecular imaging of CAF biomarkers are crucial. Platelet-derived growth factor receptor β (PDGFRβ) is highly expressed in fibroblasts, and a specific affinity probe, Z_PDGFRβ,_ that binds to PDGFRβ was successfully developed. In this study, ^47^Sc was used to label the affibody targeting PDGFRβ to explore its distribution characteristics in pancreatic cancer and the therapeutic effect of radionuclides. ^47^Sc was produced using thermal neutron irradiation-enriched ^46^Ca with a radionuclide purity greater than 99.9%. The Z_PDGFRβ_ affibody was radiolabelled with ^47^Sc to obtain a ^47^Sc-DOTA-Z_PDGFRβ_ conjugate with a radiochemical purity greater than 99%. Biodistribution studies revealed that tumour uptake of ^47^Sc-DOTA-Z_PDGFRβ_ reached 4.57 ± 2.12% ID/g at 1 h postinjection and 4.00 ± 0.71% ID/g at 96 h postinjection. However, the uptake by the liver and kidneys reached 10.44 ± 3.19% ID/g and 49.90 ± 8.89% ID/g, respectively, at 1 h postinjection. Then, the uptake was reduced to 2.20 ± 1.04% ID/g and 2.60 ± 0.27% ID/g at 96 h postinjection. Single-photon emission computed tomography (SPECT) imaging indicated specific uptake of ^47^Sc-DOTA-Z_PDGFRβ_ in PANC-2 pancreatic tumours. Similar to 177Lu, ^47^Sc exhibits an effective antitumour ability. Our results indicated that the ^47^Sc-DOTA-Z_PDGFRβ_ conjugate exhibited remarkable targeting efficacy as a PDGFRβ-targeted radiotracer in SPECT imaging and demonstrated favourable radiotherapy capabilities. SPECT imaging of ^47^Sc ions also revealed characteristic distribution patterns in the cardiac, aortic, and hepatic regions, which has significant implications for future pharmaceutical development and radiation side effect prediction.

**Supplementary Information:**

The online version contains supplementary material available at 10.1186/s12885-025-15506-w.

## Introduction

Pancreatic cancer is among the most malignant tumours and has a poor prognosis and high mortality rate [1]; it is characterized by high invasiveness, recurrence, and treatment resistance and is often accompanied by nonspecific symptoms until the tumour has developed and metastasized [2]. Patients who undergo surgical removal of local lesions exhibit an improvement in their treatment outcomes. However, for patients with regional or distant metastasis, even if timely treatment is administered, preventing disease progression remains difficult [3]. Although some patients achieve significant clinical benefits through chemotherapy, treatment resistance can still occur [4]. The microenvironment surrounding cancer cells is a crucial determinant of tumour growth and metastasis potential as well as treatment resistance [5]. Therefore, the study of the tumour microenvironment is beneficial for the precise treatment of tumours. The tumour microenvironment of pancreatic cancer is composed mainly of the extracellular matrix (ECM), vascular system and cancer-associated fibroblasts (CAFs) [6]. Changes in the histological characteristics of pancreatic carcinoma include the proliferation of connective tissue, with the fibrotic response being induced by excessive fibroblast activity and extracellular matrix deposition [7]. Fibroblasts are present in large quantities in almost every solid organ. Fibroblasts not only provide structural support but also play a significant role in the occurrence, development and metastasis of tumours [8]. CAFs modify the tumour microenvironment by reconstituting the extracellular matrix, promoting angiogenesis, and producing soluble factors that induce fibrosis, such as pathogens and damage-associated molecular patterns (PAMPs and DAMPs), cytokines (IL-6, IL-11, IL-13, IL-21, and TGF-β1), chemokines (MCP-1 and MIP-1a and b), angiogenic factors (VEGF), and growth factors (PDGF), thereby leading to cancer [9, 10].

Given the close association between CAFs and tumour progression, it is highly important to develop probes targeting CAFs and utilize molecular imaging techniques to noninvasively and precisely detect CAFs. These probes can then be applied in the diagnosis and treatment of pancreatic cancer. Several biomarkers related to CAFs have been identified, including fibroblast activation protein (FAP), α-smooth muscle actin (α-SMA), fibroblast growth factor receptor (FGFR), and platelet-derived growth factor receptor β (PDGFRβ) [11]. In pancreatic tumours, FAP is most commonly used as a target for tumour diagnosis and treatment. Some studies have demonstrated that compared with FDG, radiolabelled FAPI positron emission tomography (PET) exhibits promising potential in cancer diagnosis [12]. Furthermore, researchers have reported that PDGFRβ is highly expressed in the CAFs of various solid tumours. The core sequence of the affibody protein used for detecting PDGFRβ was obtained by the Lindborg research team through a two-step screening method [13]. They verified the high affinity and specificity of Affibody for PDGFRβ by using techniques such as SPR (Surface Plasmon Resonance), dot-blot, and FCM (flow cytometry). Building upon this foundation, we performed amino acid sequence optimization and implemented dimerization strategies to further enhance its affinity and specificity. The expression of PDGFRβ in pancreatic and liver cancer fibroblasts was subsequently examined using methods such as Western blotting and immunofluorescence staining and the Affibodies were radiolabelled with zirconium-89 (^89^Zr), gallium-68 (^68^Ga), and copper-64 (^64^Cu), and PET scanning was performed on mice harbouring tumour xenografts to obtain high-contrast images [14–16].

The emergence of novel radionuclides facilitates personalized adjustments of radiation characteristics to optimize therapeutic benefits for specific patients. The implementation of theranostics, which involves the use of a single or a match pair of radionuclides to integrate diagnosis and therapy, has attracted increasing attention in the field of nuclear medicine [17]. Examples of radionuclides used in theranostics include ^64^Cu/^67^Cu, ^124^I/^131^I, ^68^Ga/^177^Lu, and ^99m^Tc/^188^Re. The images obtained with this approach can be used to determine the distribution characteristics of drugs and provide references for estimating therapeutic doses. Scandium-47 (^47^Sc, T_1/2_=80.2 h, E_β−_ ave = 162 keV, E_γ_=159 keV) features a suitable half-life and can emit γ-rays with favourable energy and low-energy β-rays. This finding indicates that it can be used for SPECT imaging and for tumour radiotherapy. Moreover, compared with ^177^Lu, ^47^Sc can be more easily separated from the target and can form a stable complex with DOTA derivate chelators [18]. These advantages suggest that ^47^Sc is a highly promising radionuclide for theranostics. Here, we aim to label the affibody with ^47^Sc to explore its efficacy in the visual diagnosis and treatment of pancreatic cancer.

## Materials and methods

### Production of ^47^Sc

^47^Sc was produced by irradiating a ^46^Ca target (carbonate, 50 mg, 5.8% enrichment of ^46^Ca) for 9 days at a thermal neutron flux of 1.0 × 10^14^ n/(cm^2^·s) in the high-flux test reactor (HFETR) of The First Sub-institute of Nuclear Power Institute of China. Here, the nuclear reaction is ^46^Ca(n, γ)^47^Ca$\:\underrightarrow{{\beta\:}^{-}}$^47^Sc. The irradiated target decayed for 2 days after the irradiation process was completed. The outer glass tube was broken, and the powder target material was poured out. Then, the material was dissolved in 5 mL of hydrochloric acid (3 M). Approximately 50 mg of DGA resin was added to a 1 mL polypropylene column (5.6 mm × 56 mm) using the dry packing method. The column was first washed with 10 mL of 3 M HCl at a constant flow rate of 1.0 mL/min before use. After the dissolved irradiated target solution was measured using γ-ray spectroscopy, the solution was loaded into the column with DGA resin at a constant flow rate of 0.5 mL/min. Moreover, this portion of nuclear wastewater was stored in polyethylene pipes for subsequent separation. Afterwards, the Ca in the column was rinsed with 10 mL of 3 M HCl. Next, ^47^Sc was eluted with 10 mL of 0.4 M HCl. The resulting solution was heated gently until dry and redissolved in 100 µL of 0.1 M HCl. The operation was repeated after three days, after which the 47Sc was separated and eluted.

### Production of Z_PDGFRβ_ affibody

The gene encoding Z_PDGFRβ_ was cloned and inserted into a pQE30 plasmid and transformed into *Escherichia coli* (*E. coli*) M15. A small amount of bacterial solution was added to 10 ml of Luria–Bertani fluid nutrient medium (LB, 100 µg/mL Amp+, 30 µg/mL Kana+), and the sample was incubated at 37 °C and 220 rpm overnight for amplification. Then, 10 mL of LB rich in bacteria was transferred to 1.5 L of sterilized LB (100 µg/mL Amp+, 30 µg/m Kana+), and the sample was incubated at 37 °C and 220 rpm for 4–6 h until the A600 value of the bacterial solution reached 0.6–0.8. The bacteria were induced with isopropyl-L-thio-*β*-D-galactopyranoside (IPTG, 0.1 mM) at 24 °C and 120 rpm overnight. The bacteria were collected by centrifugation at 7000 × g for 10 min and resuspended in 180 mL of lysis buffer (50 mM phosphate (pH 8.0), 300 mM NaCl, 5 mM imidazole, and 1 mM phenylmethylsulfonyl fluoride), poured into a high-pressure homogenizer and gradually increased in pressure from 20 MPa to 70 MPa to completely lyse the bacteria. High-speed centrifugation (25000 g, 4 °C, 10 min) was used and repeated three times to remove bacterial precipitates, whereas the supernatant protein solution was retained. The pH of the protein solution was adjusted to 8.0, and 2 mL of Ni-NTA was added and mixed thoroughly at 4 °C overnight. The Ni–NTA and bacterial solution mixture was slowly flowed through the chromatography column. Next, 100 mL of washing buffer (50 mM PB, pH 8.0, 300 mM NaCl, and 5 mM imidazole) was used to remove impurities from the Ni gel. Elution buffer (50 mM PB, pH 8.0, 300 mM NaCl, 300 mM imidazole) was subsequently used to slowly wash and collect the target protein until the eluate no longer showed a significant colour reaction with Coomassie blue G250. Dialysis was performed overnight in phosphate-buffered saline (PBS) solution by using a dialysis bag with a retention capacity of 3.5 kDa, and the protein was stored at -80 °C after a 0.22 μm water-based filter membrane was used for filtration and sterilization. The protein concentration was measured using Coomassie blue staining. The purity and molecular weight of Z_PDGFRβ_ were estimated by sodium dodecyl sulfate–polyacrylamide gel electrophoresis (SDS–PAGE).

### Radiosynthesis of ^47^Sc-DOTA-Z_PDGFRβ_

The radioactive labelling of Z_PDGFRβ_ was achieved through the use of the bifunctional chelator 1,4,7,10-tetraazacyclododecane-1,4,7,10-tetraacetic acid mono-N-hydroxysuccinimide ester (DOTA-NHS ester). DOTA-Z_PDGFRβ_ was produced by mixing Z_PDGFRβ_ with DOTA-NHS ester at a molar ratio of 1:10 (protein to DOTA) at room temperature for 4 h after the pH of the mixed solution was adjusted to 8.0–8.5. The unconjugated DOTA-NHS ester was removed using a PD-10 chromatographic column (GE Healthcare, CA, USA). For radiolabelling, 370 MBq of ^47^ScCl_3_ solution was added to the DOTA-Z_PDGFRβ_ solution (50 µg, 50 µL) to a final volume of 250 µL. The pH of the mixed solution was adjusted to 4, 5, 6, and 7. After incubation at 40 °C for 2 h, the radiolabelling efficacy was determined with radioinstant thin-layer chromatography (radio-iTLC) using microfiber chromatography paper as the stationary phase and 0.1 M citric acid as the mobile phase. This measurement was repeated after purification using a PD-10 column to remove the free ^47^Sc. To evaluate the stability of ^47^Sc-DOTA-Z_PDGFRβ_, a solution of ^47^Sc-DOTA-Z_PDGFRβ_ was mixed with 100 µL of PBS/FBS solution (volume ratio 1:1) and incubated at room temperature. Samples of the solution were taken, and the radiolabelling efficacy was analysed using radio-iTLC at 0 h, 24 h, 48 h, and 72 h to evaluate the stability. The partition coefficient of ^47^Sc-DOTA-Z_PDGFRβ_ was evaluated using an octanol–water system. A sample of ^47^Sc-DOTA-Z_PDGFRβ_ (74 kBq) was added to a mixture of n-octanol and PBS. The mixed solution was vortexed completely for 2 min and centrifuged (6000 g, 25 min) for phase separation. Two more groups were repeated, and the average value was taken. Then, 100 µL of the upper layer liquid (Octanol) and 100 µL of the lower layer liquid (PBS) were used, and the radioactive γ counter was used to measure the radioactive count (CPM value). The partition coefficients (Log P) were expressed as the logarithm of the ratio of the counts measured in the n-octanol phase to the counts in the saline phase. The formula is Log P = Log (C_O_/C_W_).

For comparison, we labelled DOTA-Z_PDGFRβ_ with ^177^Lu. Then, 370 MBq of ^177^LuCl_3_ solution was added to DOTA-Z_PDGFRβ_ solution (50 µg, 50 µL) with 1 M sodium acetate serving as the buffer. The pH was adjusted to 5 with the addition of 0.1 M HCl. The mixtures were incubated at 40 °C for 2 h prior to radio-iTLC.

### Biomolecular interaction assays and cellular uptake of ^47^Sc-DOTA-Z_PDGFRβ_

To examine the binding affinity of the affibody before and after radioactive labelling, a biomolecular interaction assay was conducted by layer interferometry. Considering the prohibition of radioactive experimental operations in public laboratories, the products obtained by labelling nonradioactive scandium [^45^Sc] instead of ^47^Sc and nonradioactive lutetium [^175^Lu] instead of ^177^Lu were used to evaluate the affinity of the labelled affibody. The proteinA biosensors were loaded with PDGFRβ-Fc (1 µM) in PBS by dipping into the compound solution for 7 min. Afterwards, the biosensors eliminate nonspecific binding by dipping in PBS for 5 min. The binding of the labelled Sc-Z_PDGFRβ_ to its specific receptor, PDGFRβ, was measured by immersing individual sensors in a series of protein dilutions (100, 250, 500, 1000, and 2000 nM, respectively) for 240 s to determine the association rate (ka), followed by immersion in PBS for 240 s to measure the dissociation rate (kd). The biosensors were regenerated using a glycine-hydrochloride buffer solution (0.01 M, pH 2). The affinity constant (KD) was derived using the following formula: KD = kd/ka. Similarly, the assay was conducted on the lutetium labelling product Lu-DOTA-Z_PDGFRβ_.

For the cellular uptake experiment, the PDGFRβ-positive U87-MG cells were cultured in Dulbecco’s modified Eagle’s medium (DMEM) supplemented with 10% foetal bovine serum at 37 °C in 5% carbon dioxide (CO_2_). The cells were seeded at 1 × 10^5^ cells per well in six-well plates using 2 mL of normal media and then cultured overnight for adhesion. Cells were incubated with 74 kBq of ^47^Sc-DOTA-Z_PDGFRβ_ (*n* = 3) for 1 h at 37 °C in the experimental group. In the blocking group, the unlabelled Z_PDGFRβ_ (100 molar quantities of ^47^Sc-DOTA-Z_PDGFRβ_) used as the inhibitor of the labelled precursor and 74 kBq of ^47^Sc-DOTA-Z_PDGFRβ_ were simultaneously added, and competitive binding was performed at 37 °C for 1 h. In the control group, an equal volume of PBS was added as a blank. After the incubation time was reached, the culture medium containing radioactive isotopes was carefully removed from the wells using a pipette. The cells were subsequently washed 3 times with PBS and then lysed with 0.5 mL of NaOH (1 M). The cell lysate was transferred into a radioimmunoassay tube, and the radioactive γ counter was used to measure the radioactive count. The uptake rate was calculated as a percentage of the applied dose.

### Immunohistochemical and immunofluorescence assays

To detect the colocalization of PDGFRβ in tumour tissues, pancreatic cancer and paracancerous tissues were obtained from the Biobank of West China Hospital under the guidance of the Ethics Committee of the Institute (Approval No. 2024 − 640). Clinical specimens were fixed with polyformaldehyde, embedded in paraffin, and then sectioned into 20 μm slices. The antibodies used included rabbit anti-mouse PDGFRβ (Abcam, MN, USA), goat antihuman PDGFRβ (R&D, MN), HRP-conjugated goat anti-rabbit IgG, and HRP-conjugated donkey anti-goat IgG (ZenBio, Chengdu, China).

For immunofluorescence staining, PANC-2 tumour grafts were sectioned into 6 μm slices under frozen conditions, followed by incubation with a Cy3-labelled antibody against PDGFRβ (Abcam, MN, USA) at 37 °C for 1.5 h. Subsequently, the tissues were washed with PBS and incubated with the corresponding secondary antibodies (Biolegend, CA, USA) for an additional 0.5 h prior to observation under a Zeiss LSM800 laser scanning confocal microscope (Zeiss, Germany). The nuclei of the cells were visualized with diamidino-2-phenylindole (DAPI).

### Biodistribution studies

The animal studies were approved by the Animal Welfare and Ethics Committee of West China Hospital of Sichuan University (2021073 A) and were conducted in accordance with the institutional ethics committee guidelines on animal welfare. All C57BL/6 mice (4–5 weeks) were purchased from Beijing HFK Bioscience Co., Ltd. (Beijing China) and housed in specific pathogen-free (SPF)-grade barrier facilities at The Experimental Animal Center of West China Hospital, Sichuan University. C57BL/6 mice were inoculated with PANC-2 pancreatic cancer cells (5 × 10^6^ cells in 100 µL of PBS) into the subcutis of the right thigh. Biodistribution studies were performed approximately 18–21 days after cell inoculation. To evaluate whole-body biodistribution, 740 kBq of ^47^Sc-DOTA-Z_PDGFRβ_ was injected into PANC-2 pancreatic tumour-bearing mice (*n* = 3) via the tail vein.The mice used for biodistribution were anaesthetized by CO₂ inhalation prior to experimental procedures, and anaesthesia was confirmed by cervical dislocation.The animals were subsequently sacrificed at the indicated time points (1, 4, 24, 48 and 96 h). Blood, heart, liver, spleen, lung, kidney, stomach, intestine, muscle, bone, brain and tumour tissues were collected and weighed. The CPM values were determined using a radioactive γ counter. After attenuation correction, the injection dose rate per gram of each tissue and organ, expressed as %ID/g, was calculated. The formula is as follows: % ID/g = (radioactivity count per gram of organ/radioactivity count of total injected drug in the mouse).

### SPECT/CT imaging

During the SPECT/CT imaging experiment, the mice were administered 2% isoflurane in combination with oxygen at a flow rate of 1–2 L/min to ensure that the mice remained under anaesthesia. PANC-2 pancreatic tumour-bearing mice were injected with 3.7 MBq via the tail vein and then imaged using a U-SPECT small-animal SPECT/CT scanner (Millab B.V., Houten, the Netherlands). For a comparative experiment, similar procedures were performed to inject an equal amount of ^47^ScCl_3_ into another group of mice. The weight, radioactivity, and injection time of the mice were recorded. At 4 h and 24 h postinjection, the mice were anaesthetized in advance and placed on the scanning bed. SPECT/CT consisted of 240 projections, steps of 1.5°, step and shoot mode, 25 s per step with a 50 kV tube voltage and 0.2 mA tube current. Reconstruction of the SPECT images was performed with the 3D-OSEM method, after which the images could be presented in the transverse, coronal and sagittal positions.

### Radiotherapy and toxicity assessments

PANC-2 pancreatic tumour-bearing mice were randomly divided into three groups and injected with different amounts of ^47^Sc-DOTA-Z_PDGFRβ_ through the tail vein. In the low-dose group, each mouse was injected with 11.1 MBq, whereas each mouse in the high-dose group was injected with 37 MBq. Moreover, the mice in the control group were injected with an equal volume of PBS. To better evaluate the therapeutic effect of ^47^Sc on tumours, the same dose of ^177^Lu-labelled precursor, namely, ^177^Lu-DOTA-Z_PDGFRβ_, was injected into three other groups of mice as a control. The long and short diameters of the tumours were measured using a Vernier calliper, and the body weight was recorded every two days for a total of 20 days. 20 days. The tumour volume was calculated by the following formula: tumour volume (mm^3^) = (length × width × width)/2. The mice were photographed every four days to observe the trend of tumour changes. Finally, the tumour was removed and photographed. To evaluate the toxicity of radiotherapy, haematoxylin and eosin (HE) staining of the liver and kidneys of the mice was performed after the end of radiotherapy.

## Results

### Specific overexpression of PDGFR*β* in pancreatic cancer

**Fig. 1:**
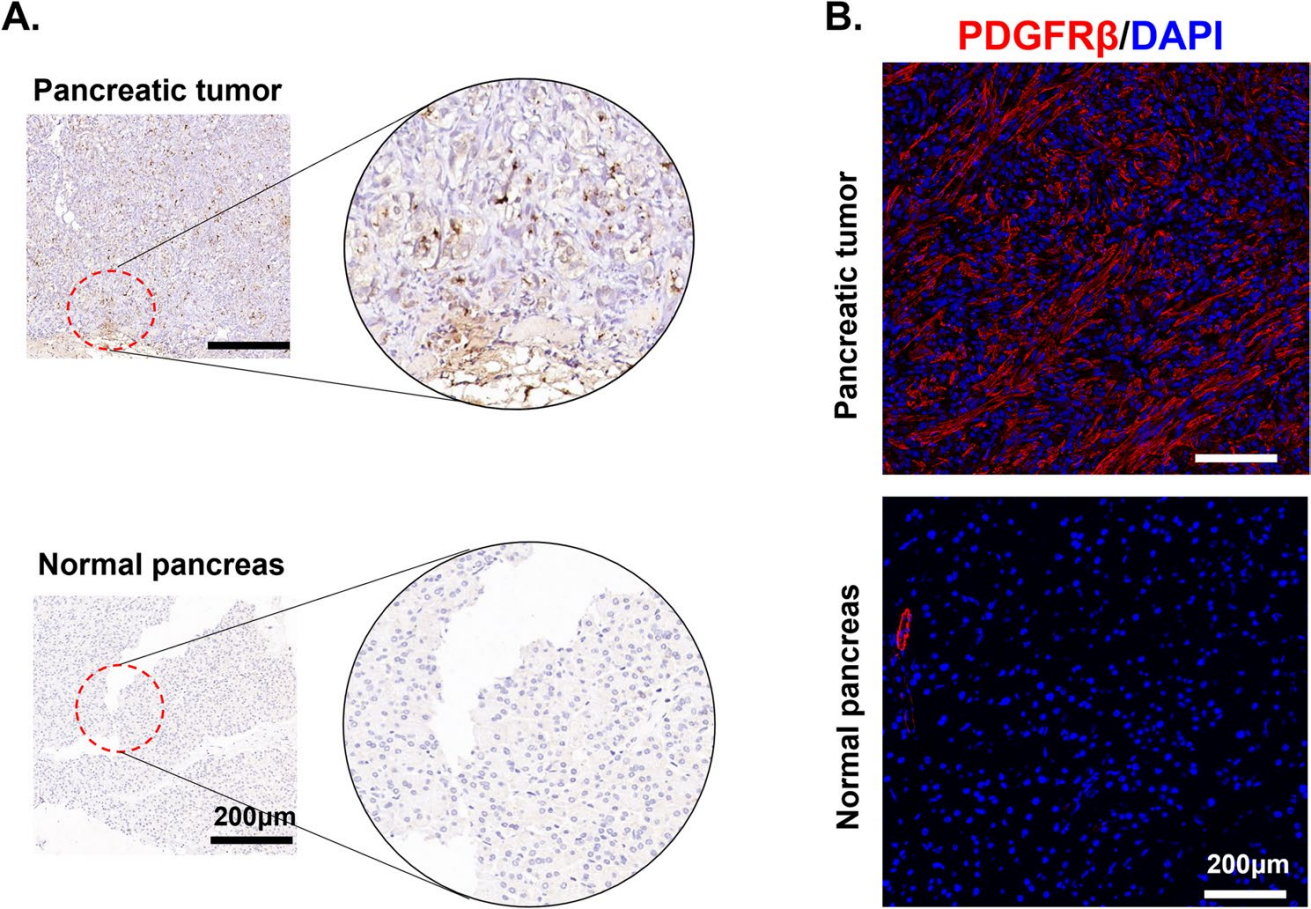
**A** Immunohistochemical staining of PDGFRβ in human pancreatic carcinoma tissues. **B** Immunofluorescent staining of PANC-2 subcutaneous tumours, and normal pancreas in mice

PDGFRβ expression in human pancreatic cancer tissues was analysed using immunohistochemical staining. As shown in Fig. 1A, characteristic PDGFRβ overexpression was observed in PDAC clinical specimens compared with that in paracancerous pancreatic tissue samples. In dissected PANC-2 tumour grafts, PDGFRβ colocalized with tumour stroma, indicating that PDGFRβ was specifically expressed in the tumour tissues of the rodent mouse model (Fig. 1B). These results suggest that PDGFRβ could be a promising molecular target for the molecular imaging of pancreatic cancer.

### Radiosynthesis of ^47^Sc-DOTA-Z_PDGFRβ_

^47^Sc was produced by the nuclear reaction of ^46^Ca(n, γ)^47^Ca$\:\underrightarrow{{\beta\:}^{-}}$^47^Sc. The irradiated ^46^Ca target was dissolved in hydrochloric acid (5 mL) and ^47^Sc was isolated using a DGA resin column. Afterwards, the ^47^ScCl_3_ solution was obtained with a radiochemical purity of 99.96% according to the gamma-ray spectrum (Figure S1).

**Fig. 2:**
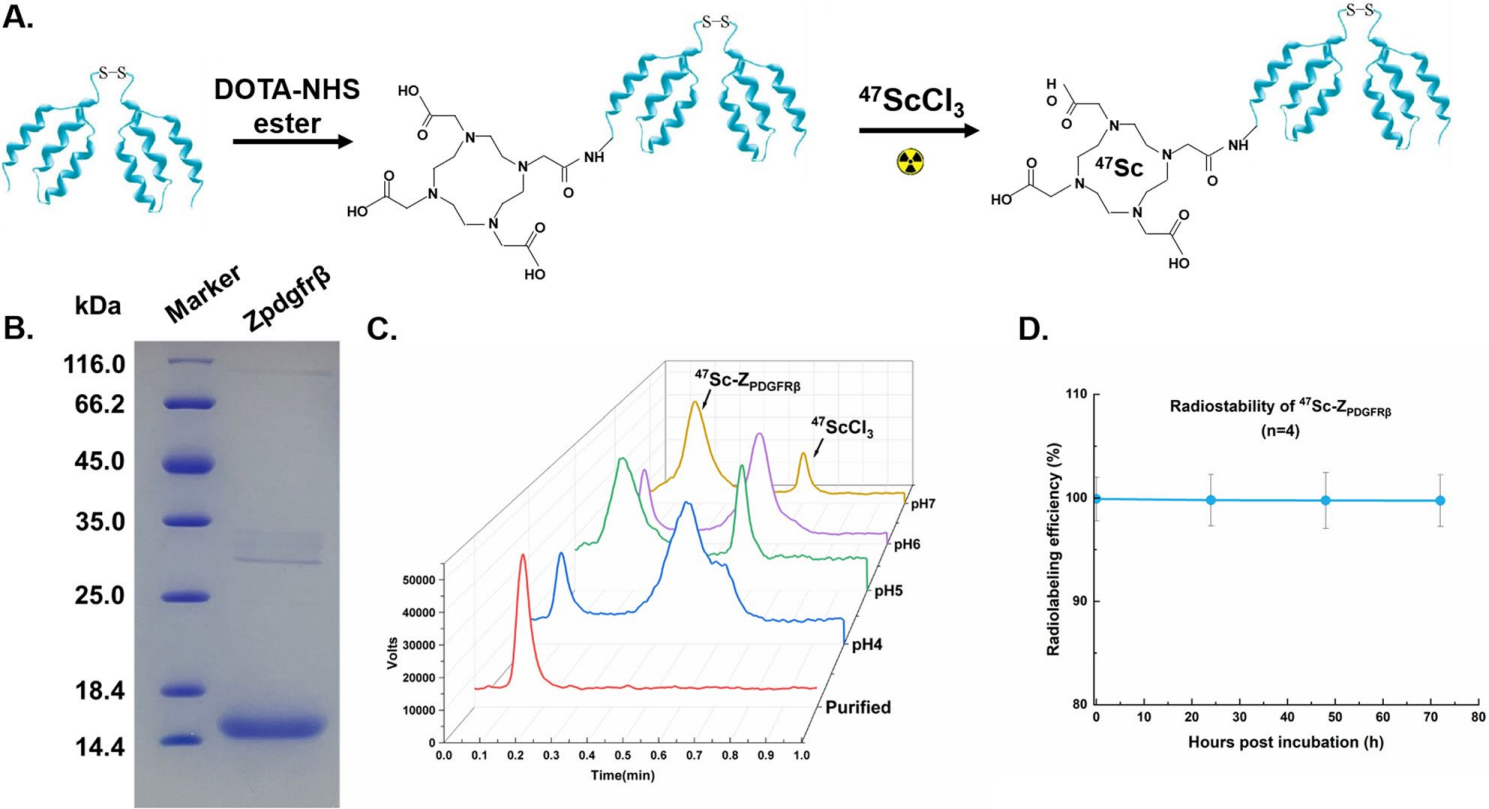
**A** Schematic diagram for the preparation of ^47^Sc-DOTA-Z_PDGFRβ_. **B** The SDS-PAGE analysis revealed that the molecular weight of Z_PDGFRβ_ is approximately 15 kDa with high purity and a singular polymeric form. **C** The labelling efficiency of ^47^Sc-DOTA-Z_PDGFRβ_ after purified and in different pH without being purified. **D** The stability assessment of purified ^47^Sc-DOTA-Z_PDGFRβ_ over a 72-hour period at room temperature conditions demonstrated its superior stability characteristic

A schematic of the radiosynthesis of ^47^Sc-DOTA-Z_PDGFRβ_ is shown in Fig. 2A. Our research team successfully prepared a Z_PDGFRβ_ affibody molecule capable of binding to PDGFRβ through recombinant expression in *E. coli*. Gel filtration chromatography revealed that the molecular weight of Z_PDGFRβ_ was approximately 15 kDa (Fig. 2B). Z_PDGFRβ_ was coupled with the bifunctional chelator DOTA. High-purity DOTA-Z_PDGFRβ_ was obtained after passing through a desalination column to remove unconjugated DOTA. To produce ^47^Sc-DOTA-Z_PDGFRβ_, the ^47^Sc solution was incubated with the DOTA-Z_PDGFRβ_ conjugate. Tests for the radioactive nuclide labelling of the DOTA-drug conjugates were subsequently conducted within a pH range spanning from 4 to 7. The results showing the influence of pH on the radiolabelling efficiency of ^47^Sc-DOTA-Z_PDGFRβ_ are presented in Fig. 2C. The quality control obtained through thin-layer chromatography indicated that the Rf value of the ^47^Sc-DOTA-Z_PDGFRβ_ product ranged from 0 to 0.2, whereas the Rf value of the unlabelled ^47^Sc product ranged from 0.5 to 0.7. The radiochemical yield was 73.38 ± 10.30% (*n* = 4) at the optimal pH of 5, and a radiochemical purity of 99.9% was achieved after PD-10 column purification. The obtained specific activity of ^47^Sc-Z_PDGFRβ_ was approximately 270 MBq after purification for each radiolabelled reaction. Notably, increased radioactivity at the original site was observed when the reaction pH was 7. This disguised peak was related to the formation of Sc(OH)_3_ precipitates in neutral-pH environments. As shown in Fig. 2D, ^47^Sc-DOTA-Z_PDGFRβ_ exhibited excellent stability in a PBS/FBS solution. After 72 h at room temperature, the radiochemical purity remained > 95%, indicating ideal stability (*n* = 4). The logP value of ^47^Sc-DOTA-Z_PDGFRβ_ was − 1.61 ± 0.03, indicating that ^47^Sc-DOTA-Z_PDGFRβ_ is slightly hydrophilic. A similar radiolabelling process was conducted for ^177^Lu-DOTA-Z_PDGFRβ_, and the radiochemical yield exceeded 95%. All the radioactive tracers were passed through a new 0.22 μm syringe filter before further use.

### Biomolecular interaction assays and cellular uptake of ^47^Sc-DOTA-Z_PDGFRβ_

**Fig. 3:**
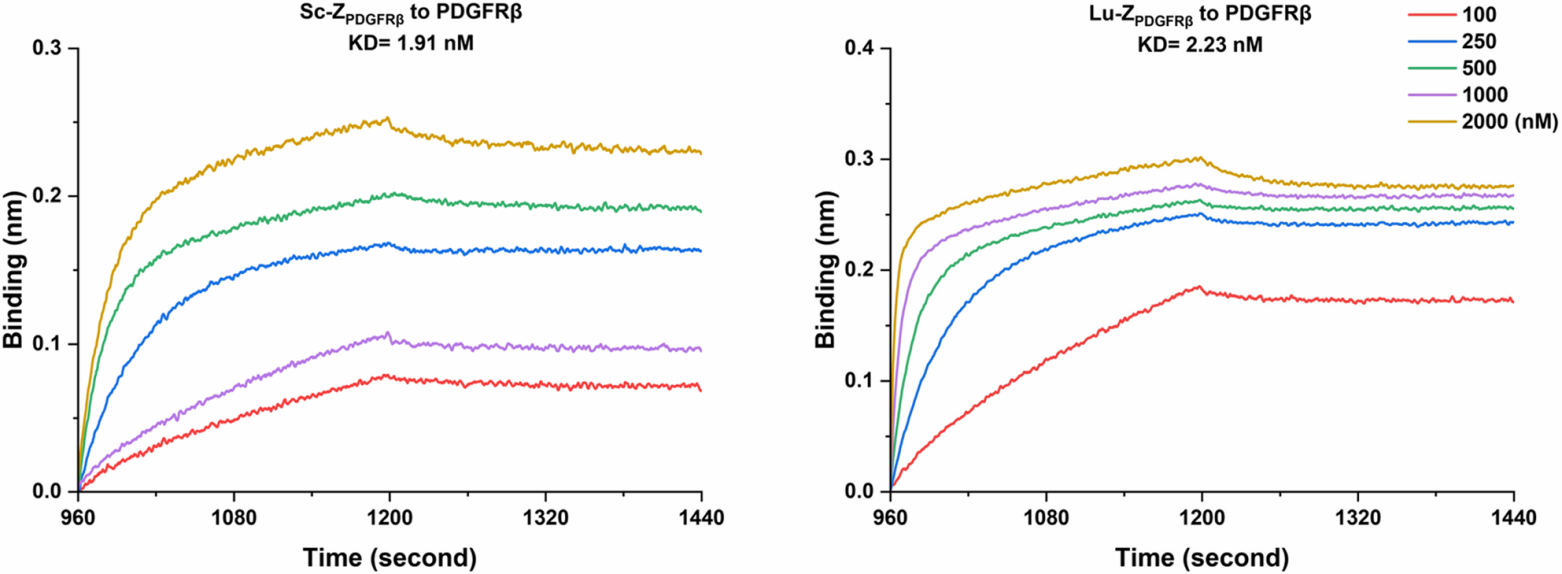
The biomolecular interaction assay was employed to measure the association and dissociation of different concentrations of Sc-Z_PDGFRβ_ and Lu-DOTA-Z_PDGFRβ_ with PDGFRβ. After fitting the curves, the obtained values of KD were 1.91nM and 2.23nM respectively

Biomolecular interaction assays provided sensorgrams showing the physical interaction of and dissociation between the affibody and the specific receptor PDGFRβ protein. As shown in Fig. 3, the obtained values of KD were 1.91nM and 2.23nM respectively which indicated that the affinity of Sc-Z_PDGFRβ_ and Lu-DOTA-Z_PDGFRβ_ for PDGFRβ was favourable.

**Fig. 4:**
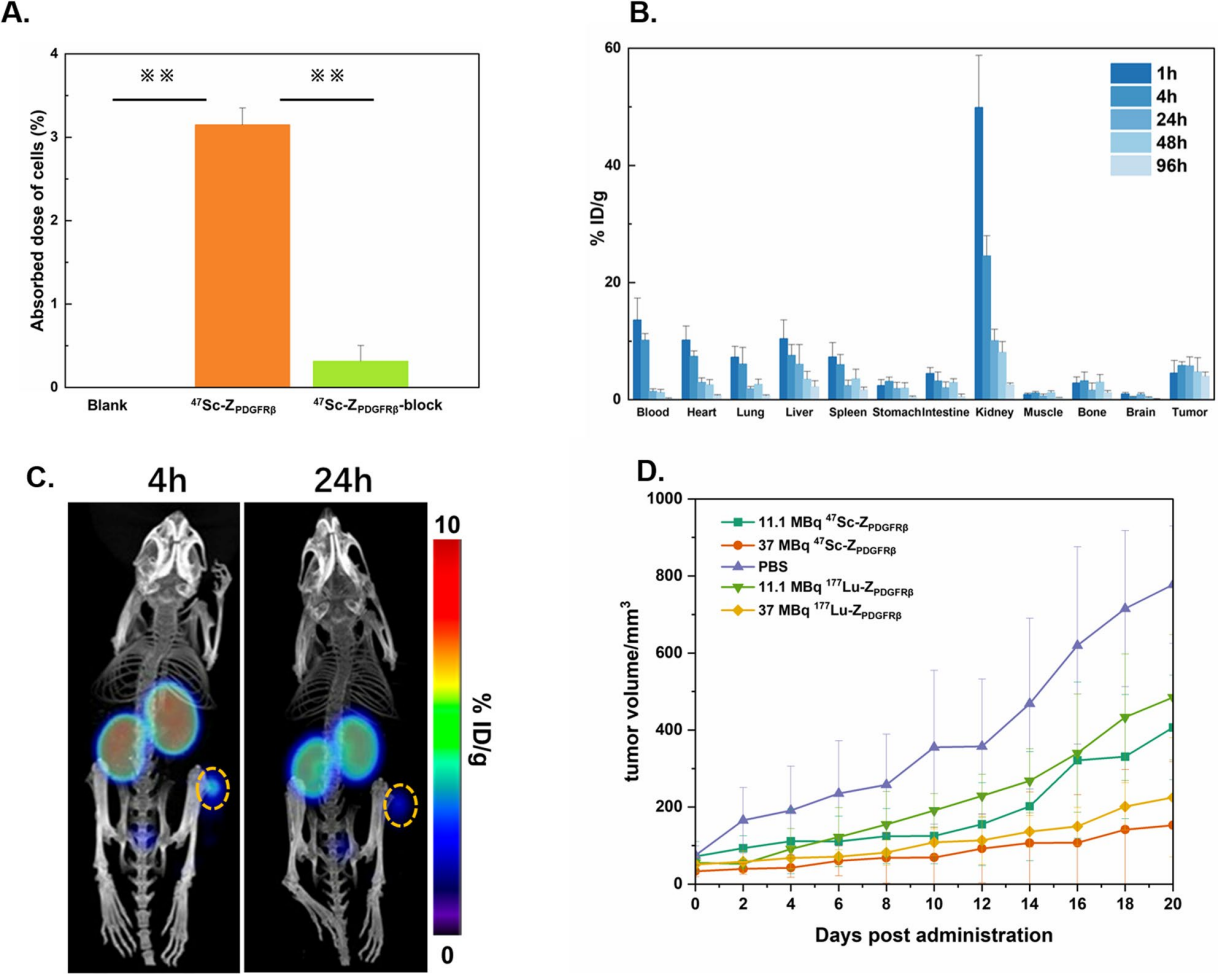
**A** Cellular binding capacity of ^47^Sc-DOTA-Z_PDGFRβ_ to U87MG cells at 1 h demonstrated that ^47^Sc-DOTA-Z_PDGFRβ_ exhibited significant binding affinity towards U87MG cells through its interaction with PDGFRβ. The statistical analysis was calculated and compared. ※*p*<0.05,※※*p*<0.01, ※※※*p*<0.001. ns: no significant (**B**) The biodistribution of ^47^Sc-DOTA-Z_PDGFRβ_ was evaluated in mice bearing PANC-2 tumours. Three mice were administered ^47^Sc-DOTA-Z_PDGFRβ_ (740 kBq) via injection, and major organs and tissues were harvested at various time points post-injection. Radioactivity was measured using a gamma counter, and tissue samples were subsequently weighed. **C** SPECT/CT imaging was performed on mice bearing PANC-2 tumours at 4-hour and 24-hour intervals following administration of ^47^Sc-DOTA-Z_PDGFRβ_, with the tumour regions delineated by yellow circles. **D** The changes in tumour volume during the treatment period in the five groups of mice (PBS, 11.1 MBq ^47^Sc-DOTA-Z_PDGFRβ_, 37 MBq ^47^Sc-DOTA-Z_PDGFRβ_, 11.1 MBq ^177^Lu-DOTA-Z_PDGFRβ_, 37 MBq ^177^Lu-DOTA-Z_PDGFRβ_)

To evaluate the binding capacity of ^47^Sc-DOTA-Z_PDGFRβ_ to the PDGFRβ protein, a cell binding assay was conducted on U87MG cells with positive PDGFRβ expression. As shown in Fig. 4A, the total uptake of ^47^Sc-DOTA-Z_PDGFRβ_ after 1 h of incubation was 3.15 ± 0.20% of the added activity, whereas the total uptake of the blocking group was only 0.31 ± 0.19% of the added activity. ^47^Sc-DOTA-Z_PDGFRβ_ has a significant binding capacity to U87MG cells (*p*<0.01), and this binding capacity could be inhibited by an excess of unlabelled DOTA-Z_PDGFRβ_. This assay demonstrated that the binding of ^47^Sc-DOTA-Z_PDGFRβ_ to U87MG cells was achieved through the interaction between Z_PDGFRβ_ and PDGFRβ [19, 20].

### Biodistribution and SPECT imaging of ^47^Sc-DOTA-Z_PDGFRβ_

As shown in Fig. 4B, the kidneys and tumours showed significant uptake of the drug in the biodistribution study. The detailed biodistribution data are presented in Table S1, and the tumour-to-organ ratios for better analysis of radionuclide retention in tumours are presented in Table S2. The biodistribution of ^47^Sc-DOTA-Z_PDGFRβ_ was characterized by rapid clearance from the blood, heart, liver, spleen, lungs and kidneys over time, indicating that the urinary-bladder system was the major excretion pathway. However, the tumour exhibited stable uptake. The tumour-to-organ ratio also tended to gradually increase over time. The accumulation of radioactivity in the tumour was 4.57 ± 2.12% ID/g at 1 h postinjection and 5.83 ± 0.65% ID/g and 5.78 ± 1.53% ID/g at 4 h and 24 h, respectively. It remained at 4.00 ± 0.71% ID/g until 96 h. The uptake in the kidneys was 49.90 ± 8.89% ID/g at 1 h after injection but decreased to 10.07 ± 1.95% ID/g at 24 h, indicating that excessive radioactive ligands were excreted through the urinary system. To evaluate the SPECT imaging feasibility of ^47^Sc-DOTA-Z_PDGFRβ_, mice bearing PANC-2 xenografts were administered ^47^Sc-DOTA-Z_PDGFRβ_, which was approximately 0.68 µg protein for each mouse, and SPECT scanning was performed at 4 h and 24 h after injection (Fig. 4C). SPECT imaging of mice confirmed the ability of ^47^Sc-DOTA-Z_PDGFRβ_ to visualize pancreatic tumour grafts.

### Antitumour study

To evaluate the therapeutic effect of the radionuclide, 11.1 MBq and 37 MBq of ^47^Sc-DOTA-Z_PDGFRβ_ were injected via the caudal vein in the PANC-2 tumour transplantation model. As a control, other groups were also injected with PBS and the same dose of ^177^Lu-DOTA-Z_PDGFRβ_ simultaneously. After continuous measurement for 20 days, we found that the tumour volume of the mice that received only PBS continued to increase over time. However, in the mice treated with ^47^Sc-DOTA-Z_PDGFRβ_, tumour growth clearly significantly delayed in a dose-dependent manner, which was similar to the tumour growth trend in the mice treated with ^177^Lu. Compared with that in the group that received only PBS, the tumour volume in the 11.1 MBq and 37 MBq ^47^Sc-DOTA-Z_PDGFRβ_ groups decreased by 48% and 80%, respectively, 20 days after administration, whereas the tumour volume in the 11.1 MBq and 37 MBq ^177^Lu-DOTA-Z_PDGFRβ_ groups decreased by 38% and 71%, respectively. These results indicate that the effects of ^47^Sc were similar to those of ^177^Lu (Fig. 4D). During the treatment period, no significant changes in the body weight of the mice were observed, and no deaths occurred. HE staining of organs after treatment revealed that the structures of the liver and kidneys were clear. These findings indicated that the therapeutic doses of ^47^Sc-DOTA-Z_PDGFRβ_ and ^177^Lu-DOTA-Z_PDGFRβ_ are nontoxic to the mice (Figure S3).

### Influence of pH on scandium forms

**Fig. 5:**
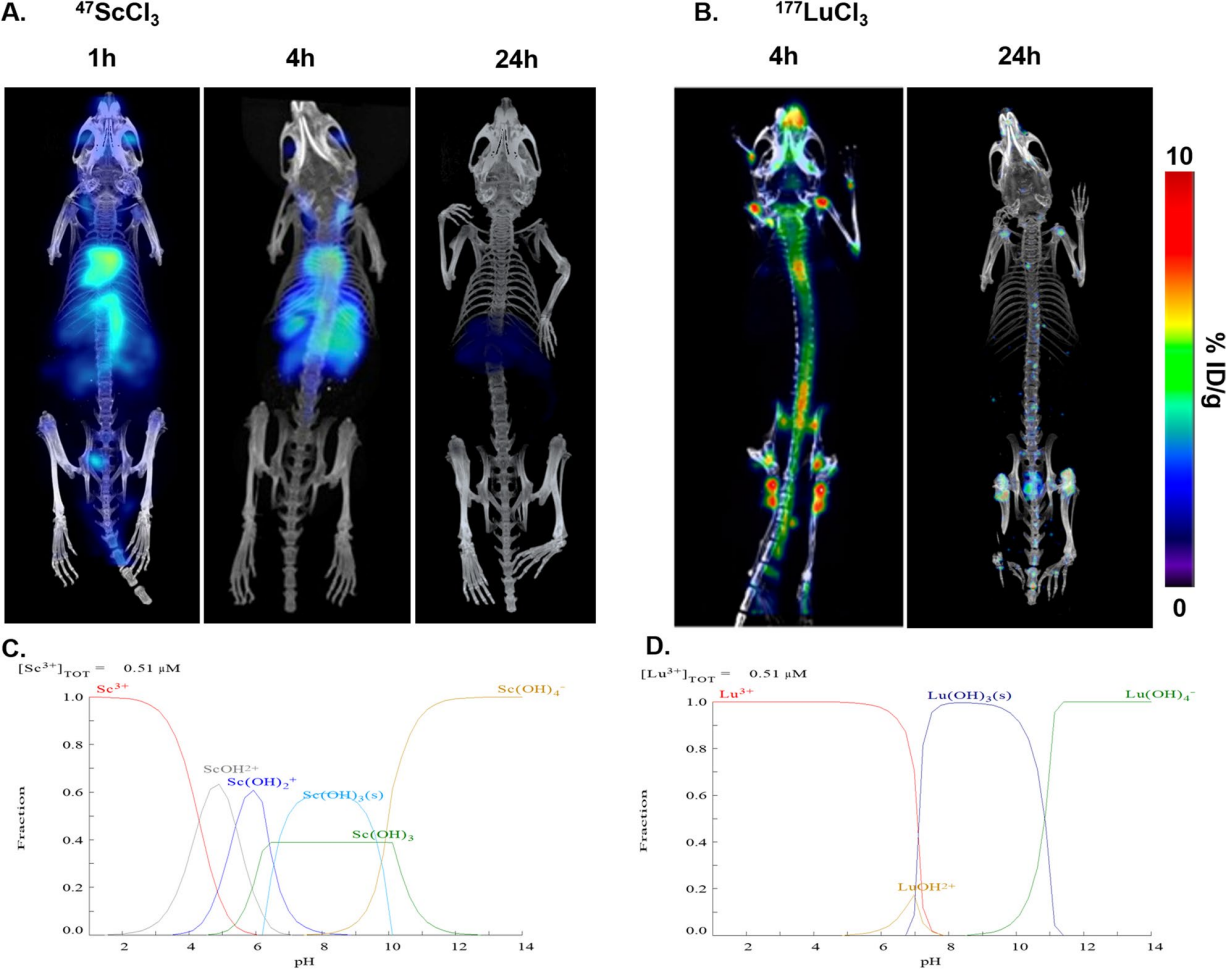
**A** SPECT/CT imaging of ^47^ScCl_3_ in mice. **B** SPECT/CT imaging of ^177^Lucl_3_ in mice. **C** Calculated precipitation equilibrium of scandium (III) at different pH values. **D** Calculated precipitation equilibrium of scandium (III) at different pH values. Symbol “(s)” represented an unknown crystal form precipitate

In contrast, SPECT imaging of ^47^ScCl_3_ was conducted in tumour-bearing mice (Fig. 5A). ^47^ScCl_3_ was distributed mainly in the heart, abdominal aorta and liver at 1 h and 4 h. Twenty-four hours later, it was distributed mainly in the liver and intestine and then entered the intestinal excretory system through the bile duct. This was different from the obvious bone affinity characteristic of the previous ^177^LuCl_3_ solution on SPECT/CT (Fig. 5B). Moreover, ^47^ScCl_3_ biodistribution was evaluated in mice bearing PANC-2 tumours (Figure S2). In the biodistribution analysis, significant uptake of free ^47^Sc^3+^ was consistently observed in the blood, heart, and liver. Furthermore, progressive increases in radiotracer accumulation were demonstrated in both hepatic and intestinal tissues over time, which was consistent with the changing pattern of the SPECT image. We predicted the speciation changes of scandium and lutetium nuclides at different pH values using MEDUSA software [21] and determined that the pH value may be the cause of this difference. As illustrated in Fig. 5C, a significant amount of insoluble Sc(OH)_3_ formed in the solution when the pH exceeded 6. Consequently, ^47^ScCl_3_ formed ^47^Sc(OH)_3_ precipitates in the neutral environment in the body, and the radioactive precipitates were deposited in the heart, abdominal aorta and liver and circulated through the body’s circulatory system (Fig. 5A). This observation also aligned with the results obtained from our labelling experiment, which demonstrated an increase in the radioactive peak of radioactive precipitates at Rf = 0–0.2 when the reaction pH = 7 (Fig. 2C). However, Lu^3+^ remains in a free ionic state during systemic circulation when the pH is below 7.6 (Fig. 5D). Free Lu³⁺ ions are primarily taken up by the reticuloendothelial system, such as the liver and spleen, and secondarily by osteoblasts. Owing to the similar chemical properties between Lu³⁺ and calcium ions, Lu³⁺ preferentially deposits on the surface of hydroxyapatite in bones, especially in areas with active bone metabolism, which leads to bone marrow suppression [22]. Therefore, the free Lu³⁺ showed clear bone uptake on the SPECT image (Fig. 5B).

## Discussion

Cancer-associated fibroblasts (CAFs) are among the principal cellular components in various malignant solid tumours. They not only promote the production of the extracellular matrix but also facilitate angiogenesis and generate soluble factors capable of inducing fibrosis. Together with the extracellular matrix, CAFs constitute the tumour microenvironment, which influences the progression, invasion, metastasis, and therapeutic resistance of tumour cells [23]. Therefore, the use of CAFs represents a crucial strategy for tumour diagnosis and treatment. Among several biomarkers associated with CAFs, such as fibroblast activation protein (FAP), α-smooth muscle actin (α-SMA), fibroblast growth factor receptor (FGFR), and platelet-derived growth factor receptor β (PDGFRβ), FAP stands out. Recent studies have also indicated that PDGFRβ serves as a crucial oncogenic regulator of cancer cell migration, proliferation, and angiogenesis through pathways such as stimulating fibroblast chemotaxis and activating the EGFR signalling pathway [24]. PDGFRβ is highly expressed in liver cancer, colon cancer, liver fibrosis, and pancreatic cancer tissues. Z_PDGFRβ_ affibody molecules that exhibit high affinity for PDGFRβ have been successfully expressed in *E. coli*. Affibody molecule proteins are advantageous because of their small molecular weight, low immunogenicity, favourable folding, high tolerance to modification, high tissue permeability, good stability, robust affinity, and favourable pharmacokinetics [25], increasing their potential as carriers for cytotoxic drugs in cancer treatment [26] and making them attractive in the field of radiodiagnosis and radiotherapy [27]. Through the process of radiolabelling this probe with appropriate radioactive nuclides and subsequent PET/CT and SPECT/CT scans, we obtained high-quality contrast images. These images exhibit distinct and clear visualization, offering crucial and invaluable references for accurate diagnosis. This accomplishment further solidifies the hypothesis that PDGFRβ can be effectively exploited as a viable target for both diagnostic and therapeutic strategies in the context of pancreatic cancer. These results not only enhance our understanding of the role of PDGFRβ in pancreatic cancer but also provide a strong impetus for the development of more targeted and effective diagnostic and treatment modalities. In this study, we labelled Z_PDGFRβ_ with ^47^Sc to obtain the radioactive conjugate ^47^Sc-DOTA-Z_PDGFRβ_.

^47^Sc emits low-energy gamma rays, making it suitable for SPECT/CT imaging. Additionally, it can generate beta rays, which are used for targeted radionuclide therapy. The half-life (T_1/2_ = 80.2 h) is moderate; thus, it is suitable for long-distance transportation, allowing imaging to longer time points [28] and ensuring radiation safety for patients. ^47^Sc has similar chemical properties to Y^3+^ and Lu^3+^. Like these compounds, scandium exists almost entirely in the trivalent state. Consequently, the ligands designed for these cations are likely to be equally applicable for chelating Sc [29]. Among the various chelating agents, DOTA is the most appropriate for scandium. DOTA can rapidly form a stable complex with scandium from a thermodynamic perspective and demonstrates kinetic inertness [30]. We investigated the influence of pH on radiolabelling. This is because scandium belongs to the transition metal group, and the formation of its complexes strongly depends on the pH of the aqueous solution. The pH value is an important parameter in the optimization process of radiolabelling [31]. The efficacy of radioactive labelling demonstrated a regular pattern in response to pH variations. Under strongly acidic conditions, its efficacy was notably lower.

The macrocyclic chelator DOTA forms highly stable complexes with numerous trivalent radionuclides used in molecular imaging or therapy, whether in its free form or conjugated with targeting molecules [32]. In this study, cell binding experiments demonstrated that the binding affinity of U87MG cells to ^47^Sc-DOTA-Z_PDGFRβ_ was significantly enhanced, suggesting the potential of utilizing Z_PDGFRβ_ for tumour imaging by targeting PDGFRβ in pancreatic cancer. SPECT/CT images of ^47^Sc-DOTA-Z_PDGFRβ_ clearly revealed the tumour grafts in the subcutaneous tumour model mice. Moreover, as time progressed, the tumour still exhibited stable uptake of the radioactive tracer. This finding was consistent with the changing trend of tumours in radioactive biological distribution studies. Notably, in the radiobiological distribution assay, the ^47^Sc signal in the kidneys rapidly accumulated initially but then gradually decreased. These findings suggest that the conjugate was excreted via the urinary system. The results of the partition coefficient assay also indicated that the conjugate was hydrophilic. These findings suggest that considering the radiation safety of the kidneys during subsequent radionuclide therapy is essential. Several methods can be used to overcome this limitation by modifying the tracer [33, 34]. One viable approach is to fuse Z_PDGFRβ_ with an albumin-binding domain through genetic modification or direct chemical modification. On the basis of our most recent research findings, the addition of an albumin-binding domain not only reduces renal accumulation but also prolongs the in vivo circulation of the drug and enhance drug uptake by tumours. The albumin fusion strategy can effectively improve the in vivo pharmacokinetics of various radiolabelled antibodies and small-molecule drugs. Human serum albumin (HSA), a natural endogenous drug carrier, is less likely to induce immune rejection [35]. The affibody-albumin complex formed after binding to HSA has a molecular weight exceeding the glomerular filtration threshold, and the steric hindrance effect upon binding can shield the Megalin binding sites on the affibody molecule surface, effectively reducing renal active uptake. Furthermore, binding to albumin facilitates FcRn recycling, which maintains blood drug concentration homeostasis and enhances drug accumulation at the lesion site [36]. A series of small-molecule EB-conjugated drugs and ABD-amino acid-fused Her2-targeted affibodies, such as ^177^Lu-EB-PSMA [37], ^99m^Tc-ADAPT6 [38], ^177^Lu-ABY-027 [34], and ^177^Lu-PEP49989 [39], have been developed in recent years.

In addition to molecular imaging, Z_PDGFRβ_ plays a crucial role in targeted radionuclide therapy (TRT) for pancreatic cancer. To more comprehensively evaluate the radiotherapy efficacy of ^47^Sc, we selected ^177^Lu, which has similar chemical properties to ^47^Sc and is increasingly used in radiotherapy with a well-established approach, as a reference. In the experimental therapy study, tumour growth clearly depended on dose. In the high-dose group (37 MBq), the tumour grew slowly, and the tumour volume was smaller than that in the low-dose group (11.1 MBq). However, no difference was observed in the tumour growth curves between the ^47^Sc-DOTA-Z_PDGFRβ_ and ^177^Lu-DOTA-Z_PDGFRβ_ groups at the same dose, indicating that the tumour radiotherapy effects of ^47^Sc are similar to those of ^177^Lu. Moreover, its ability to enable SPECT/CT imaging will offer more ideal options for the integrated and precise diagnosis and treatment of tumours using the same radionuclide.

The majority of adverse effects of radiopharmaceuticals are primarily derived from radionuclide dissociation from their carrier compounds, leading to free form and circulation within the body. However, the potential hazards of ^47^Sc have not been evaluated in past studies. In this study, the accumulation of radioactivity in the blood pool was observed, and chemical precipitation equilibrium provided a possible explanation for this phenomenon. Previous research has indicated that the chemical properties of complexes, including lattice energy, solvation energy, and complex stability constants, are strongly correlated with the size of metal ions. For complexes formed with Sc^3+^, which is smaller than Lu^3+^, the coordination numbers and geometric structures of the two ions demonstrate both similarities and differences [40]. Overall, the pH-sensitive precipitation equilibrium of scandium ions requires alternative perspectives for evaluating the side effects and optimizing the radiolabelling procedures of ^44^Sc/^47^Sc radiopharmaceuticals.

## Conclusions

In this study, we constructed the affibody probe Z_PDGFRβ_, which targets PDGFRβ, and successfully radiolabelled it with ^47^Sc. We evaluated the imaging effect of ^47^Sc via visual diagnosis and verified the specific targeting ability of the affibody probe Z_PDGFRβ_. Moreover, we found that similar to ^177^Lu, ^47^Sc has favourable tumour radiotherapy efficacy. Our research results indicated that ^47^Sc-DOTA-Z_PDGFRβ_ is valuable for achieving the integration of targeted radiodiagnosis and radiotherapy. This study also marked the initial discovery of the in vivo imaging characteristics of free ^47^Sc ions. These findings indicate that in future applications of radioisotope therapy, we need to pay attention to radiation safety issues in these tissues and organs.

## Supplementary Information

Supplementary Material 1.

Supplementary Material 2.

Supplementary Material 3.

## Data Availability

All data generated or identified data supporting the findings of this study are available from the corresponding author upon reasonable request.
