# Supplementary material for: Investigation of a 47Sc-radiolabelled PDGFRβ-targeted affibody in SPECT imaging and radiotherapy for pancreatic cancer
Source: BMC Cancer. 2026 Jan 5;26:168. doi: 10.1186/s12885-025-15506-w (PMC12870327; doi:10.1186/s12885-025-15506-w)
Supplement: Supplementary file 1 — Supplementary Material 1. [file 12885_2025_15506_MOESM1_ESM.docx]

**Full-length original image of Figure 2(B)**


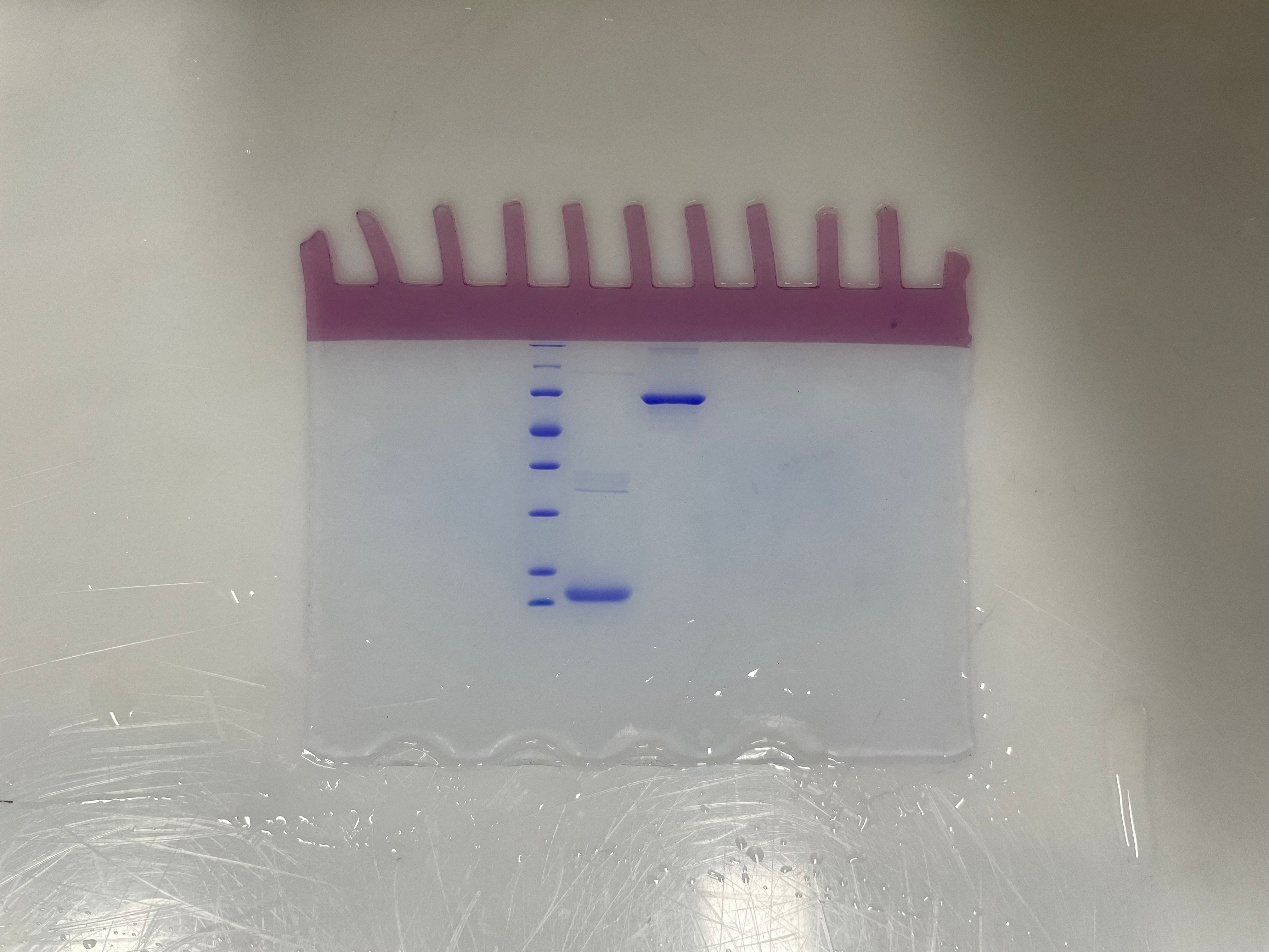


From left to right:

(1) Marker

(2) Z_PDGFRβ_: 15 kDa

(3) Bovine Serum Albumin (BSA) : 66.4 kDa, 0.5 mg/mL

This gel picture was taken on a white plastic background board under LED lighting in the laboratory by a mobile phone. This gel was uncropped and the picture was not processed.


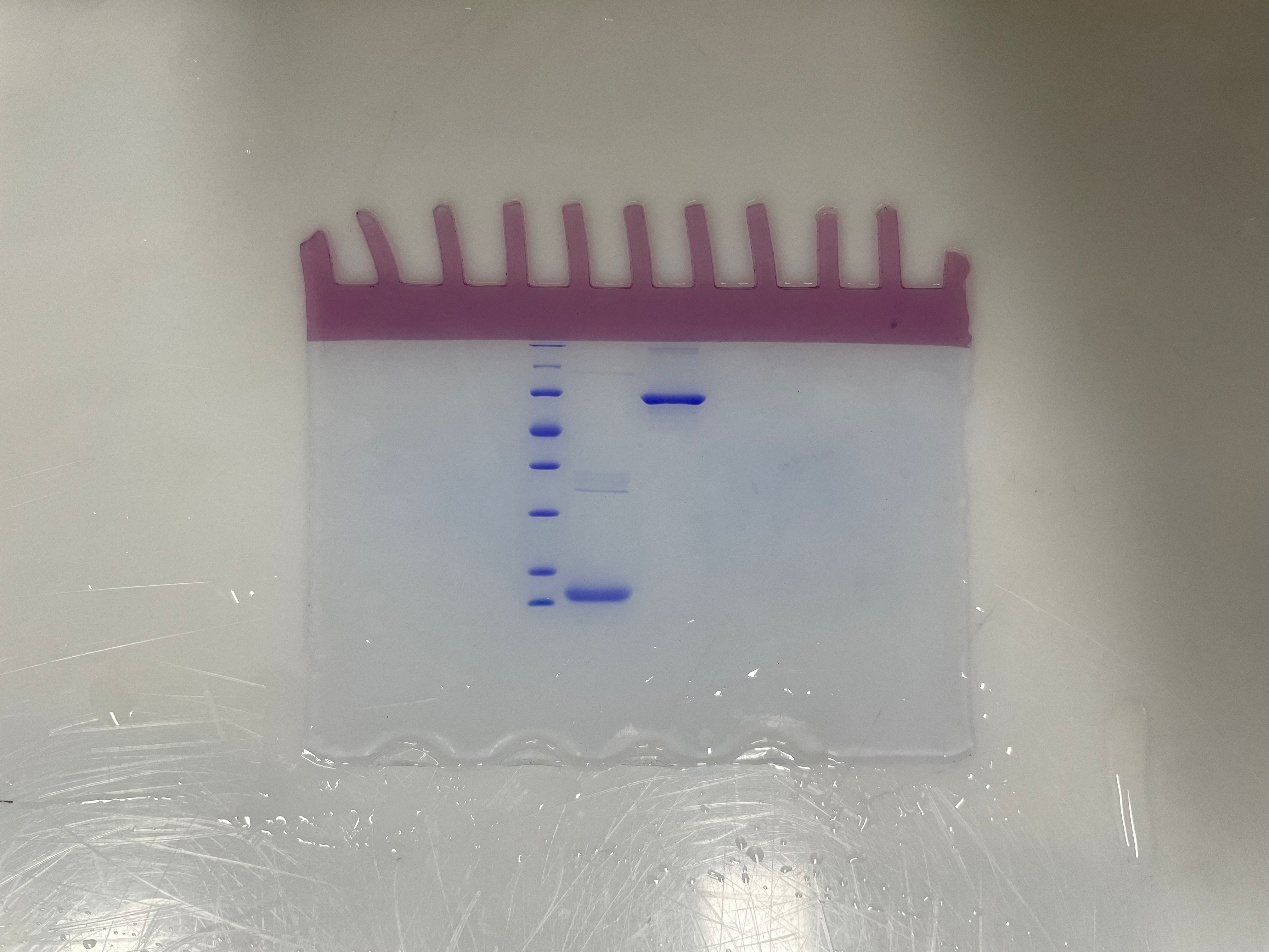


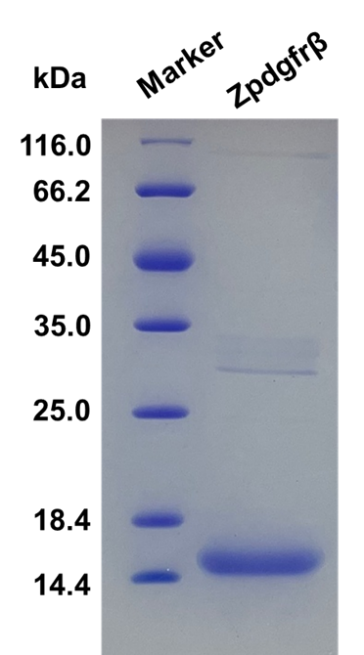


The area indicated by the red square frame represents Figure 2(B) in the manuscript. And the molecular weight of each protein of the marker was shown on the left side. The SDS-PAGE analysis revealed that the molecular weight of Z_PDGFRβ_ is approximately 15 kDa with high purity and a singular polymeric form.
