# Supplementary material for: Investigation of a 47Sc-radiolabelled PDGFRβ-targeted affibody in SPECT imaging and radiotherapy for pancreatic cancer
Source: BMC Cancer. 2026 Jan 5;26:168. doi: 10.1186/s12885-025-15506-w (PMC12870327; doi:10.1186/s12885-025-15506-w)

**Figure S1.** The γ-ray spectrum of ^47^Sc obtained from irradiated ^46^CaCO_3_ targets




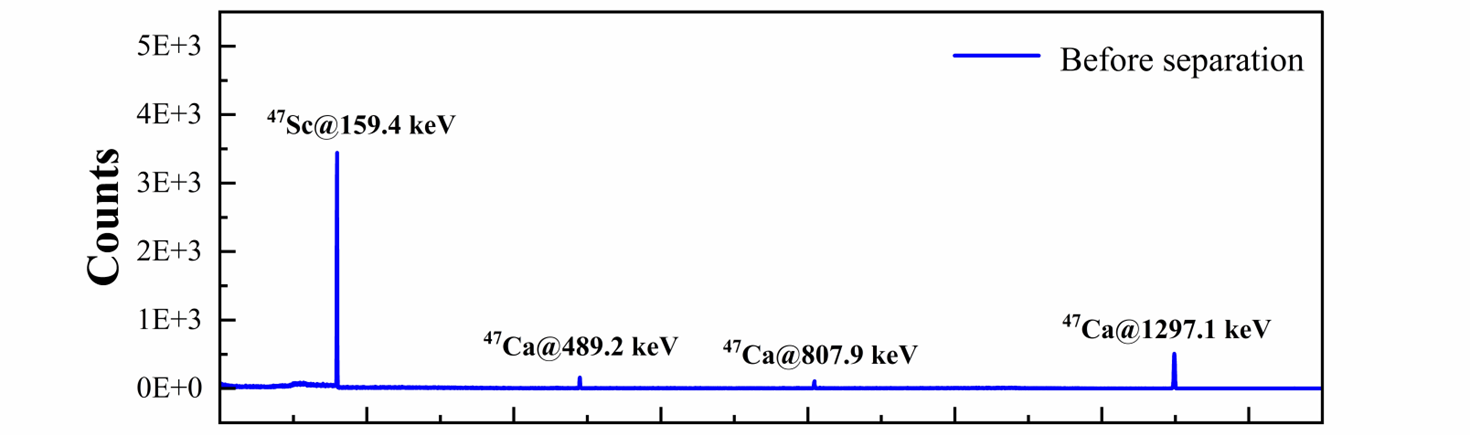

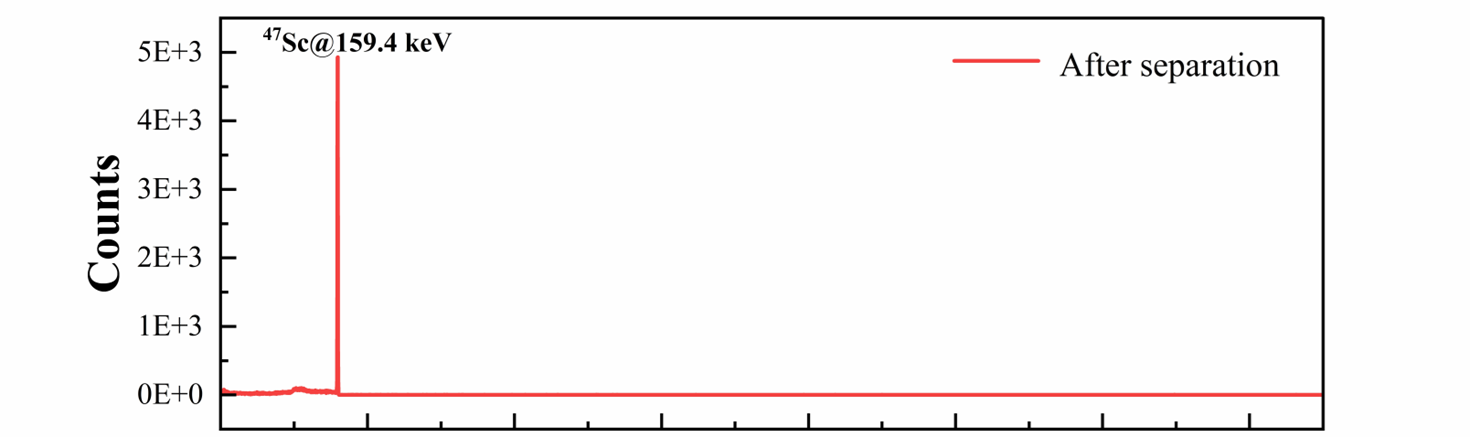


**Figure S2.** The biodistribution of ^47^ScCl_3_ was evaluated in mice bearing PANC02 tumours. Three mice were administered ^47^ScCl_3_ (740 kBq) via injection, and major organs and tissues were harvested at various time points post-injection. Radioactivity was measured using a gamma counter, and tissue samples were subsequently weighed.





**Figure S3.** HE staining of liver and kidney from three groups of mice (N=5) showed the structures were clear


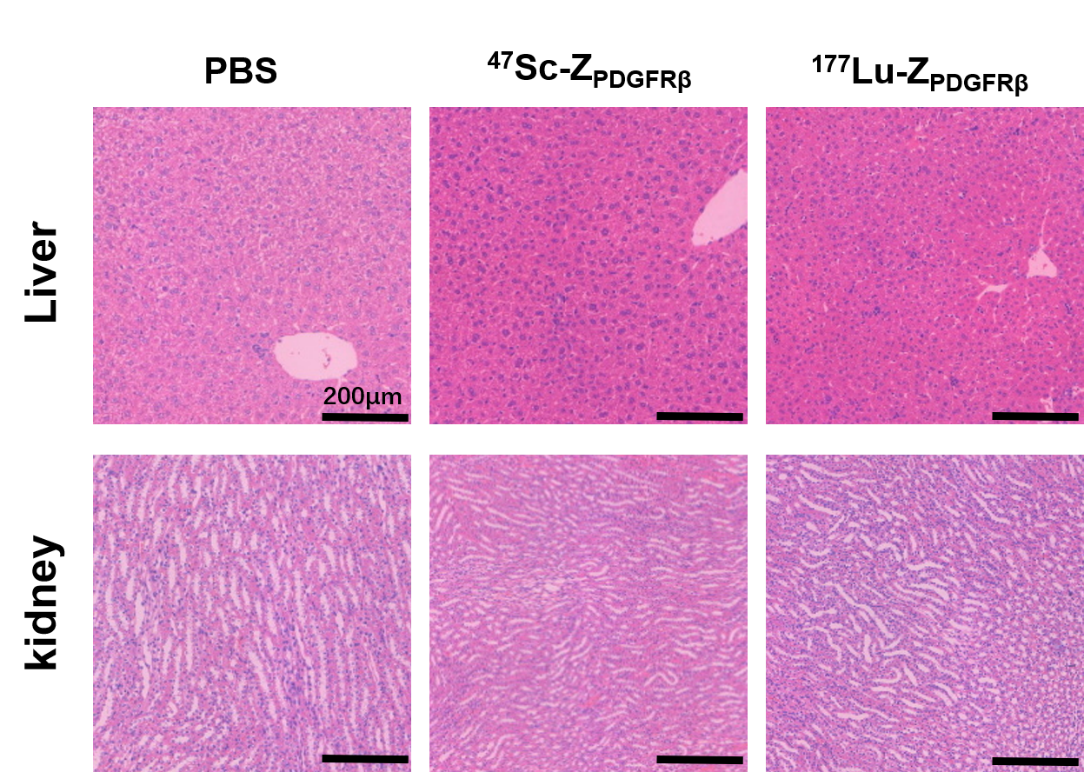

Supplement: Supplementary file 2 — Supplementary Material 2. [file 12885_2025_15506_MOESM2_ESM.docx]
