# Supplementary material for: Investigation of a 47Sc-radiolabelled PDGFRβ-targeted affibody in SPECT imaging and radiotherapy for pancreatic cancer
Source: BMC Cancer. 2026 Jan 5;26:168. doi: 10.1186/s12885-025-15506-w (PMC12870327; doi:10.1186/s12885-025-15506-w)
Supplement: Supplementary file 3 — Supplementary Material 3. [file 12885_2025_15506_MOESM3_ESM.docx]

**Table S1**. Biodistribution of ^47^Sc-DOTA-Zpdgfrβ in PANC-2 pancreatic tumour bearing mice (n=3) at 1h, 4h, 24h, 48h and 96h

| Organ \ Time | 1h | 4h | 24h | 48h | 96h |
| --- | --- | --- | --- | --- | --- |
| Blood | 13.63±3.73 | 10.16±1.11 | 1.42±0.43 | 1.24±0.50 | 0.21±0.12 |
| Heart | 10.19±2.38 | 7.39±0.91 | 2.97±0.73 | 2.54±0.89 | 0.77±0.10 |
| Lung | 7.29±1.82 | 6.08±2.86 | 1.87±0.38 | 2.63±0.87 | 0.68±0.13 |
| Liver | 10.43±3.19 | 7.59±1.82 | 6.05±3.36 | 3.52±1.30 | 2.20±1.04 |
| Spleen | 7.29±2.45 | 5.99±1.72 | 2.41±0.94 | 3.59±1.61 | 1.66±0.47 |
| Stomach | 2.42±1.02 | 3.14±0.70 | 1.92±1.04 | 1.96±0.93 | 0.49±0.15 |
| Intestine | 4.47±1.01 | 3.18±1.53 | 2.04±0.99 | 2.92±0.65 | 0.51±0.46 |
| Kidney | 49.90±8.89 | 24.53±3.47 | 10.07±1.95 | 8.09±1.82 | 2.60±0.27 |
| Muscle | 0.93±0.21 | 1.15±0.26 | 0.65±0.32 | 1.24±0.28 | 0.34±0.05 |
| Bone | 2.87±1.02 | 3.25±1.47 | 1.65±1.23 | 3.01±1.27 | 1.19±0.38 |
| Brain | 0.98±0.24 | 0.53±0.04 | 0.93±0.23 | 0.37±0.12 | 0.12±0.07 |
| Tumour | 4.57±2.12 | 5.83±0.65 | 5.78±1.53 | 4.77±2.39 | 4.01±0.71 |

Data are presented as average %ID/g and SD

**Table S2.** Ratio of tumour to organ from the biodistribution of ^47^Sc-DOTA-Zpdgfrβ in PANC-2 pancreatic tumour bearing mice (n=3) at 1h, 4h, 24h, 48h and 96h

| Ratio=Tumour/Organ | 1h | 4h | 24h | 48h | 96h |
| --- | --- | --- | --- | --- | --- |
| Blood | 0.34±0.10 | 0.58±0.07 | 4.09±0.15 | 5.20±4.16 | 22.68±7.60 |
| Heart | 0.49±0.27 | 0.80±0.13 | 2.07±0.79 | 2.28±1.56 | 5.34±1.18 |
| Lung | 0.61±0.12 | 1.09±0.39 | 3.06±0.18 | 1.98±0.98 | 5.96±1.03 |
| Liver | 0.42±0.05 | 0.79±0.10 | 1.08±0.30 | 1.52±0.80 | 2.03±0.65 |
| Spleen | 0.63±0.13 | 1.01±0.14 | 2.86±1.46 | 1.62±1.06 | 2.57±0.79 |
| Stomach | 2.25±1.34 | 1.91±0.34 | 3.57±1.28 | 3.63±3.18 | 8.42±1.45 |
| Intestine | 1.07±0.45 | 2.09±0.71 | 3.32±1.61 | 1.70±0.74 | 11.98±5.78 |
| Kidney | 0.09±0.04 | 0.24±0.03 | 0.57±0.10 | 0.60±0.24 | 1.56±0.28 |
| Muscle | 4.98±1.69 | 5.18±0.66 | 10.15±3.00 | 4.27±2.49 | 11.74±1.86 |
| Bone | 1.58±0.26 | 1.99±0.55 | 5.00±3.19 | 1.85±1.08 | 3.49±0.51 |
| Brain | 4.50±0.78 | 11.2±1.64 | 6.70±2.49 | 15.16±9.73 | 36.98±9.96 |
